# Degradation of Bunker C Fuel Oil by White-Rot Fungi in Sawdust Cultures Suggests Potential Applications in Bioremediation

**DOI:** 10.1371/journal.pone.0130381

**Published:** 2015-06-25

**Authors:** Darcy Young, James Rice, Rachael Martin, Erika Lindquist, Anna Lipzen, Igor Grigoriev, David Hibbett

**Affiliations:** 1 Biology Department, Clark University, Worcester, MA, United States of America; 2 School of Engineering, Brown University, Providence, RI, United States of America; 3 Joint Genome Institute, Walnut Creek, CA, United States of America; Genetics and Microbiology Research Group, SPAIN

## Abstract

Fungal lignocellulolytic enzymes are promising agents for oxidizing pollutants. This study investigated degradation of Number 6 “Bunker C” fuel oil compounds by the white-rot fungi *Irpex lacteus*, *Trichaptum biforme*, *Phlebia radiata*, *Trametes versicolor*, and *Pleurotus ostreatus* (Basidiomycota, Agaricomycetes). Averaging across all studied species, 98.1%, 48.6%, and 76.4% of the initial Bunker C C10 alkane, C14 alkane, and phenanthrene, respectively were degraded after 180 days of fungal growth on pine media. This study also investigated whether Bunker C oil induces changes in gene expression in the white-rot fungus *Punctularia strigosozonata*, for which a complete reference genome is available. After 20 days of growth, a monokaryon *P*. *strigosozonata* strain degraded 99% of the initial C10 alkane in both pine and aspen media but did not affect the amounts of the C14 alkane or phenanthrene. Differential gene expression analysis identified 119 genes with ≥ log_2_(2-fold) greater expression in one or more treatment comparisons. Six genes were significantly upregulated in media containing oil; these genes included three enzymes with potential roles in xenobiotic biotransformation. Carbohydrate metabolism genes showing differential expression significantly accumulated transcripts on aspen vs. pine substrates, perhaps reflecting white-rot adaptations to growth on hardwood substrates. The mechanisms by which *P*. *strigosozonata* may degrade complex oil compounds remain obscure, but degradation results of the 180-day cultures suggest that diverse white-rot fungi have promise for bioremediation of petroleum fuels.

## Introduction

White-rot basidiomycetes are primary agents of lignocellulose decay [1]. The unique suite of fungal oxidative enzymes that effects decay of the recalcitrant lignin polymer has been studied for potential uses in biofuel production and bioremediation [2]. The availability of numerous genomes of wood-rotting fungi significantly contributes to this effort [3]. Investigating gene families that contribute to decay of various substrates may uncover further biotechnology applications. White-rot basidiomycetes degrade organic pollutants such as polycyclic aromatic hydrocarbons (PAHs), explosives, polychlorinated biphenyls (PCBs), and organochlorine pesticides [4]. Fungal ligninolytic enzymes may be able to oxidize pollutants with chemical structures similar to the phenylpropane monomers of lignin molecules [5]. The fungal class II peroxidases lignin peroxidase (LiP), manganese peroxidase (MnP), and versatile peroxidase (VP) contribute to the degradation of lignin polymers via direct oxidation or non-specific oxidation with free radicals [6]. The multicopper oxidase laccase catalyzes the one-electron oxidation of diverse phenolic substrates, including the phenolic component of lignin [7]. Numerous studies have linked these enzymes to roles in pollutant breakdown [8]. Other studies suggest that cytochrome P450 monooxygenases catalyze the first step in PAH degradation [9, 10], or that PAHs with high ionization potentials are primarily oxidized by MnP-dependent lipid peroxidation reactions [11]. Alkanes, which compose 20–50% of crude oil, are oxidized in terminal oxidation reactions that are catalyzed in fungi by microsomal cytochrome P450 monooxygenases in the CYP52 family [12].

Five of the six fungal species in this study have been previously evaluated for bioremediation potential. Whole cultures and isolated enzymes from *Irpex lacteus* degrade PAHs with 3–6 rings [13], TNT [14], synthetic dyes [15, 16], and other pollutants [17, 18]. Similar studies have been conducted with *Pleurotus ostreatus* [19–21], *Trametes versicolor* [22–24], *Phlebia radiata* [25], and *Trichaptum biforme* [26, 27], but this is the first study to assess the pollutant degradation capabilities of Punctularia strigosozonata. The experimental wood-decay fungi are reported on as few as fourteen genera of host trees (P. strigosozonata) or over 50 (Irpex lacteus, Trametes versicolor) [28–30]. All six species occur most often on diverse hardwoods and can be considered hardwood specialists, but they are all rarely or occasionally reported on conifer substrates.

This study was initiated as part of an effort to develop a bioremediation strategy at Fisherville Mill, a brownfield remediation site on the Blackstone River in Grafton, MA. The heavy residual petroleum product Number 6 “Bunker C” fuel oil was used as heating fuel at a former onsite textile mill that burned down in 1999. Underground oil storage tanks were damaged in the fire and Bunker C oil leaked into surrounding soils, sediments, and surface water, including the historic Blackstone Canal. Although the US Environmental Protection Agency (EPA) conducted a cleanup of the site following the fire, surface waters remained contaminated with Bunker C oil in spring 2011 [31]. To remove Bunker C oil from canal water, a bioremediation system was constructed by John Todd Ecological Design, Inc. (Woods Hole, MA). One component of the system is a “mycofiltration” system, in which contaminated canal water is passed through bins containing wood chips inoculated with wood-decaying fungi. The potential effectiveness of this method is suggested by studies indicating the ability of various species of white-rot fungi to degrade crude oils and petroleum distillates in complex media like soil [32, 33].

The primary goal of this study was to assess the extent to which five species of white-rot fungi are able to degrade petroleum-derived hydrocarbons, which could address the potential effectiveness of the mycofiltration system and inform choices of fungal species in the bioremediation efforts at Fisherville Mill. The secondary goal was to assess gene expression in another white-rot species, *P*. *strigosozonata*, in pine and aspen sawdust media, with and without Bunker C oil.

## Materials and Methods

### Strains and growth conditions

Bunker C oil was collected from the Blackstone Canal at a brownfield remediation site in Grafton, MA, USA (42°10'39.3"N 71°41'25.8"W) with permission from the private site owner (Fisherville Redevelopment LLC). The oil was autoclaved three times at 121°C for 1h15m with 24h between each cycle.

Dikaryotic strains of *I*. *lacteus* FD-9, *P*. *radiata* FD-121, *P*. *ostreatus* FD-119, *T*. *versicolor* FP-101664 SS-1, and *T*. *biforme* FD-177 obtained from the USDA Forest Products Laboratory (Madison, WI) were maintained on malt yeast agar (MYA) plates without light at 27°C. MYA plates contained 20g/L agar (Difco, BD), 20g/L malt extract (EMD Chemicals), and 0.5g/L yeast (EM Science) in 1L water, autoclaved at 121°C for 30 minutes of sterilization time. These fungal cultures were inoculated onto media containing eastern white pine (*Pinus strobus*) sawdust as this was the principal wood type under consideration for use in the “mycofiltration” system at Fisherville Mill. Semisolid media for *I*. *lacteus*, *T*. *biforme*, *P*. *radiata*, *T*. *versicolor*, and *P*. *ostreatus* cultures was made with 600 mL wheat bran, 2400 mL *P*. *strobus* sawdust, and 1400 mL water. This amount was halved according to weight and 20 g of Bunker C oil was mixed into one half. Approximately 25 g of media was weighed into each glass Petri dish. To inoculate media containing Bunker C oil, 0.5 cm^3^ cubes of new growth were taken from MYA plates and placed in the center of each dish. Dishes were wrapped in Parafilm (Bemis Company), incubated without light at 27°C for 180 days and then stored at -20°C. The Petri dishes containing media without oil were uninoculated and kept in the same conditions as the inoculated dishes to serve as controls for the hydrocarbon degradation analysis.

### Hydrocarbon degradation analysis

Hydrocarbon degradation was measured with duplicate cultures of *P*. *ostreatus*, *T*. *biforme*, *P*. *radiata*, and *T*. *versicolor*. Degradation was measured with a single culture of *I*. *lacteus*. Remaining amounts of three Bunker C oil compounds with varying recalcitrance and toxicity were assayed in 180-day cultures by gas chromatography-mass spectrometry (GC-MS): the PAH phenanthrene, an alkane with 10 carbon atoms (C10) and an alkane with 14 carbon atoms (C14). Analysis of individual Bunker C components (rather than of total petroleum hydrocarbons) enabled comparison of the performance of the study organisms to previous research on specific compounds. The representative compounds were also measured in uninoculated controls of media without Bunker C oil to assess the signal of each compound in the media components.

Cultures were dried for 48 hours (LabConco, Model 77530–10). Oil and other soluble components were extracted from the cultures with methylene chloride by five 10 m static cycles (~1600 psi, 373°K) of accelerated solvent extraction (Dionex Model ASE 200), which is designed to remove sorbed organic compounds from strong sorbents and provides nearly complete recovery of deuterated standards. Considering the harsh ASE conditions, it is unlikely that any pollutant adsorbing to the fungal hyphae will be unaccounted for in the extract solution. Approximately 1 g of sample material was transferred from each Petri dish to a clean 34 mL stainless steel ASE capsule. Any capsule volume void of sample material was filled with clean, inert sand to reduce the total amount of solvent used during extraction. Methylene chloride was evaporated from the liquid effluent (Zymark TurboVap LV). Remaining oil residues were dissolved in methylene chloride to a total volume of 10 mL. The compositions of these analytes were acquired by GC-MS (Agilent, Models GC: HP6890; MS: 5973) using a modified U.S. EPA 8015c method [34].

GC-MS obtained peak retention times and mass spectra were compared to those of an alkane and PAH-containing standard calibration mixture. The retention times and spectra for the chosen C10 and C14 alkanes matched those of decane and tetradecane, respectively. However, the Bunker C oil used in this study was environmentally weathered and contained a complex mixture of hydrocarbons, so it was not possible to say with complete certainty that these compounds were in fact unsubstituted decane and tetradecane. Thus, they are referred to here as “C10” and “C14” alkanes.

The extent of oil degradation was estimated by comparing the ratios of three biodegradable compounds to one non-biodegradable marker in the original autoclaved oil and the experimental cultures. Hopane (C_30_H_52_), a complex triterpene with a molecular weight of 412.7 (mass to charge ratio m/z = 191, retention time = 38.3 min) was chosen as the non-biodegradable marker. Hopane and other compounds belonging to the terpenoid family have long been used as petrochemical biomarkers due to their environmental persistency and stability [35, 36] and their resistance to degradation by aerobic microbiota [37]. The C10 and C14 alkanes (m/z = 57, retention time = 10.1 and 20.4 m, respectively) and the PAH phenanthrene (m/z = 178, retention time = 20.0 m) were chosen to represent the degradable organic compounds. Chromatographic peaks were identified and integrated to calculate the area of the peak. Ratios of biodegradable compounds to the non-biodegradable marker were calculated separately for each sample. Sample ratios were divided by the compound-to-marker ratios in autoclaved oil to normalize for the concentration of oil in each sample and then converted to percentages of compounds degraded per sample:

$$\left(\frac{compound_{exp}}{hopane_{exp}}\right)/\left(\frac{compound_{oil}}{hopane_{oil}}\right)\;x\;100$$ (1)

A degradation ratio quotient of 0.5 before conversion to percentage represents a 50% degradation (by mass) of the compound. Note that the use of an internal standard to quantify the extent of biodegradation would not have been practical in this study due to the heterogeneity (oil + sawdust + fungi + moisture) of each sample and potential degradation (i.e., mass reduction) of bulk oil. In other words, though 1 g of mixed media was analyzed, the amount of bulk oil in each sample, which is needed to calculate component concentrations, was not obvious.

The quantities of the compounds of interest attributable to the wood in the spawn were not subtracted from the amounts attributed to the oil due to differences in spawn mixtures between cultures grown for 180 days and those grown for 20 days. Therefore, the actual amount of phenanthrene and C14 alkane degradation is likely being underestimated by a marginal amount.

### Gene expression analysis

Cultures in four experimental conditions (pine, pine with Bunker C oil, aspen, and aspen with Bunker C oil) were made in triplicate to analyze gene expression by the *P*. *strigosozonata* monokaryon HHB-11173 Pine media was used to approximate conditions in the “mycofiltration” system at Fisherville Mill while aspen media was used to assess potential differential gene expression in relatively extractive-free conditions, compared to pine media. Pine media for the transcriptome cultures was made with 250mL bran, 1000mL pine sawdust, and 500mL water. Semisolid aspen (*Populus tremuloides*) media for the transcriptome cultures was prepared with 125mL bran, 500mL aspen sawdust, and 500mL water. 65 g of Bunker C was mixed into 650 g of both types of media. Approximately 27.5 g of media was weighed into each glass Petri dish. Two uninoculated dishes were kept as media controls, one with Bunker C oil and one without, for GC-MS analysis. To inoculate media with *P*. *strigosozonata* HHB-11173 SS-5, 0.125 cm^3^ cubes of new mycelial growth on agar from MYA plates were placed in the center of each dish. Dishes were incubated without light at 27°C for 20 days and then stored at -80°C. Experimental and control cultures were assessed for Bunker C degradation by GC-MS as described for the hydrocarbon degradation analysis.

Total RNA was extracted from triplicate cultures of *P*. *strigosozonata* by harvesting three samples of between 0.87–0.93g that transected mycelial age (from center to edge). Samples were ground with mortar and pestle in liquid nitrogen prior to total RNA extraction with a Qiagen RNeasy Midi Kit and on-column DNase digestion. The manufacturer’s instructions were followed with the recommended modifications for tissue homogenization using a syringe and needle. The three extractions from each culture were combined before LiCl purification to meet concentration standards for Illumina sequencing (1 μg/μl total RNA). Extractions were assessed by denaturing gel electrophoresis with ethidium bromide and quantified using an MWGt LambdaScan 200 or a Shimadzu UV-1650PC UV-visible spectrophotometer.

Two biological replicates from each condition were chosen for sequencing at the Joint Genome Institute (JGI, Walnut Creek, CA) based on RNA quantity and quality (S1 Table). mRNA was purified from total RNA (Absolutely mRNA, Stratagene) and chemically fragmented to 200–250 bp (RNA Fragmentation Reagents, AM8740 –Zn, Ambion). First strand cDNA was synthesized using Superscript II Reverse Transcriptase (Invitrogen) and random hexamers. The second strand was synthesized using *E*. *coli* RnaseH, DNA ligase, and DNA polymerase I for nick translation. The dscDNA was then cleaned using a double SPRI bead selection (Agencourt Ampure beads). The TruSeq Sample Prep kit (Illumina) was used for RNASeq library creation using the manufacturer’s instructions. The second strand was removed by AmpErase UNG (Applied Biosystems). Paired end 100 bp reads were sequenced on an Illumina HiSeq 2000 for unamplified stranded TruSeq.

Reads were trimmed to 36bp and aligned to *P*. *strigosozonata* gene models with the Burrows-Wheeler Aligner (BWA) [38]. Raw mapped read counts were analyzed for differential expression in the Bioconductor package DESeq in R version 2.15.3 [39]. After libraries were normalized for size, four treatment comparisons were made: 1) aspen media + Bunker C oil vs. aspen media, 2) pine media + Bunker C oil vs. pine media, 3) pine media vs. aspen media, and 4) pine media with oil vs. aspen media with oil. A principal components analysis (PCA) was conducted in DESeq to ordinate the reads from each treatment. Transcript IDs returned by DESeq as being differentially expressed ≥ log_2_(2-fold) with an adjusted p value ≤ 0.01 were used as queries in JGI’s Mycocosm fungal genome browser (genome.jgi.doe.gov) [40], which uses the best representative gene model from the query transcript to predict functional domains from the Wellcome Trust Sanger Institute Pfam database (pfam.xfam.org) [41]. Mycocosm user annotations and Pfam domain annotations were gathered to assess categories of genes that were differentially regulated in pairs of media treatments.

## Results

Visual estimations of colony diameters revealed no apparent differences among species in growth rates on each replicate plate, with or without oil present. All species completely colonized media with and without oil by 180 days of growth.

### Hydrocarbon degradation within 180 days

**Fig 1:**
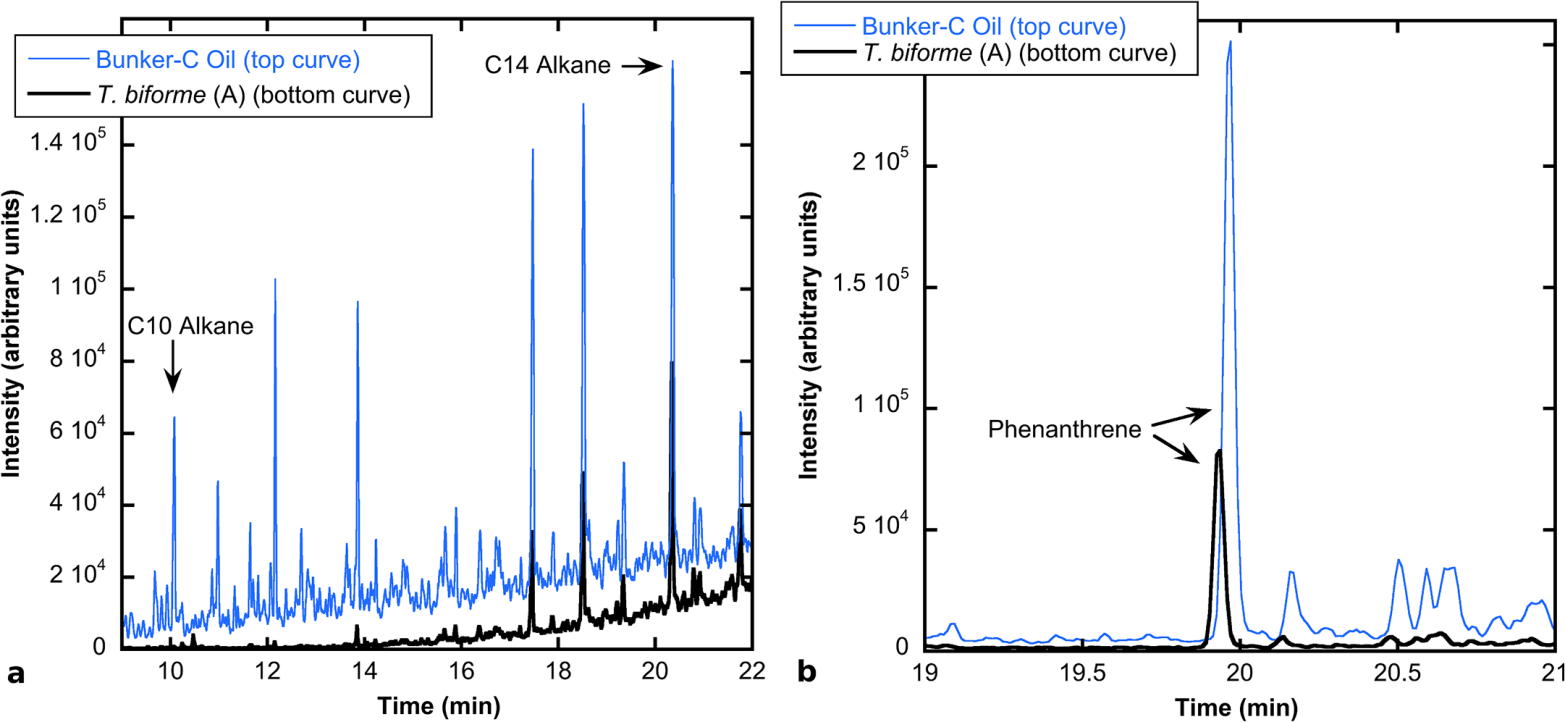
Hydrocarbon degradation by *Trichaptum biforme*. GC-MS chromatograms of (a) alkane and (b) phenanthrene degradation by *T*. *biforme* measured after 180 days of growth in pine media with Bunker C oil. Black lines = *T*. *biforme* profiles; blue lines = Bunker C oil profiles.

**Fig 2:**
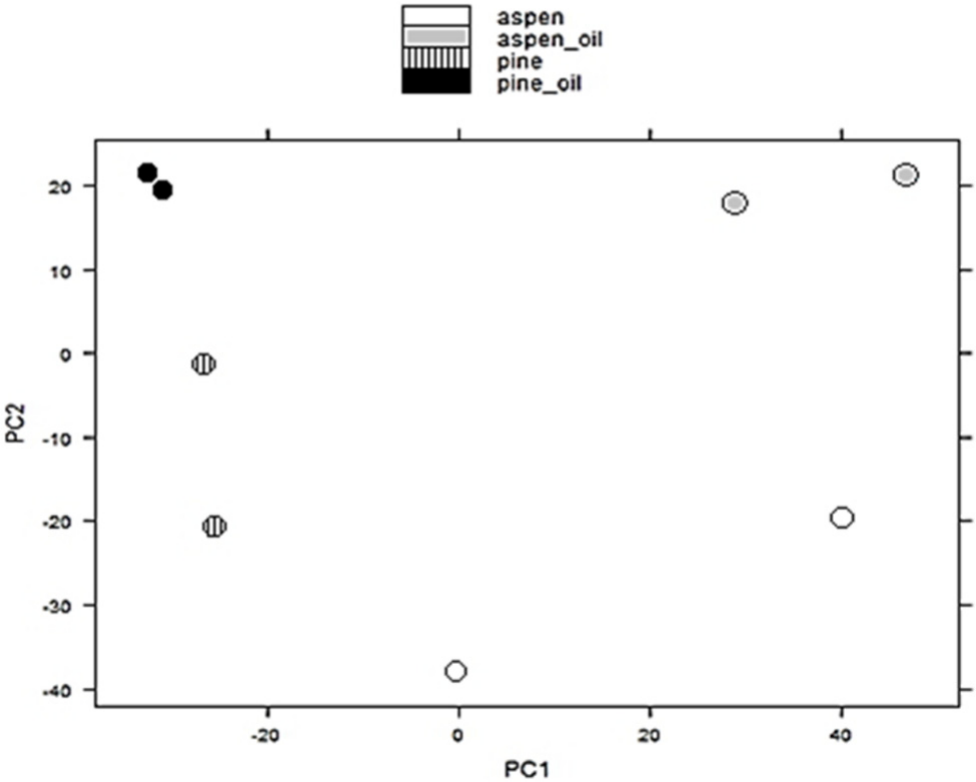
Biplot of principal components (PC) axes PC1 and PC2 derived from a Principal Components Analysis (PCA) of mapped paired-end Illumina RNA-Seq reads from 20 day-old *Punctularia strigosozonata* cultures in media treatments (aspen, aspen with Bunker C oil, pine, and pine with Bunker C oil). Ordination was completed in the R package DESeq.

Oil compound degradation was assessed by comparing peak intensities in GC-MS signals between samples and autoclaved oil (example chromatograms of *T*. *versicolor* sample A and Bunker C oil in Figs 1A and 2B). Hopane (retention time = 38.3 m) and a C10 alkane (retention time = 10 m) in Bunker C oil were not present in noteworthy amounts in spawn controls (S1 and S3 Figs). The phenanthrene (retention time = 19.9 m) and C14 alkane (retention time = 20.2 min) peaks in the oil were detected in small amounts in uninoculated media controls (S2 and S3 Figs). The phenanthrene/hopane, C14 alkane/hopane, and C10 alkane/hopane compound peak area ratios in the autoclaved Bunker C oil were used to normalize the compound/hopane ratios in the experimental cultures so that the percentages of compound degradation could be compared among samples (Eq 1; Table 1).

**Table 1:** Degradation (%) of phenanthrene, a C14 alkane, and a C10 alkane in Bunker C oil by white-rot fungi.

Sample	Phenanthrene	C14 alkane	C10 alkane
180-day cultures			
*I*. *lacteus*	76.9%	54.4%	98.0%
*P*. *radiata* (A)	89.7%	61.3%	99.0%
*P*. *radiata* (B)	66.7%	46.3%	99.0%
*P*. *ostreatus* (A)	84.6%	35.5%	97.0%
*P*. *ostreatus* (B)	94.9%	48.1%	98.0%
*T*. *versicolor* (A)	84.6%	46.3%	98.0%
*T*. *versicolor* (B)	43.6%	34.5%	96.0%
*T*. *biforme* (A)	74.4%	54.7%	99.0%
*T*. *biforme* (B)	71.8%	56.4%	99.0%
Mean (180 day cultures)	76.4%	48.6%	98.1%
20-day cultures			
*P*. *strigosozonata* (pine)	-2.6%	-5.2%	99.0%
*P*. *strigosozonata* (aspen)	5.1%	-7.0%	99.0%
Mean (20 day cultures)	1.3%	-6.1%	99.0%

Degradation percentages were calculated using Eq 1.

All cultures grown for 180 days reduced the amount of C10 alkane in pine spawn cultures with oil relative to the amount in pure autoclaved oil by at least 96% (Table 1). The C14 alkane decreased by an average of 48.6% and phenanthrene decreased by an average of 76.4% across all cultures and biological replicates (Table 1).

Degradation of the C10 alkane between pairs of biological species replicates was similar while reproducibility of phenanthrene and C14 alkane degradation was low between duplicates (Table 1).

### *P*. *strigosozonata* hydrocarbon degradation and gene expression after 20 days

The *P*. *strigosozonata* monokaryon strain HHB-11173 SS-5 degraded a C10 alkane in Bunker C oil by 99% on pine and aspen media within 20 days (Table 1). Phenanthrene and a C14 alkane were resistant to degradation in the 20-day growth period, although *P*. *strigosozonata* degraded 5.1% of the original phenanthrene in aspen media (Table 1).

RNA-Seq reads of *P*. *strigosozonata* grown on pine and aspen media with and without Bunker C oil mapped to the *P*. *strigosozonata* genome (≥91%) and transcriptome (≥78%) (Table 2). Information about RNA purity and concentration is presented in S1 Table. Principal components analysis and Pearson correlations revealed that mapped RNA-Seq reads in biological duplicate cultures grown on pine spawn with Bunker C oil were highly correlated (r = 0.98) while reads in the remaining conditions exhibited more variation between duplicates (pine media r = 0.85, aspen media r = 0.76, aspen with oil media r = 0.73) (Fig 2). The first principal component appears to correspond to wood species used in sawdust media, whereas the second principal component corresponds to the presence or absence of Bunker C oil.

**Table 2:** Illumina RNA-Seq read counts and percentages of reads mapped to the *Punctularia strigosozonata* genome and transcriptome.

			% of reads mapped	
Growth media	Replicate	RNA-Seq read count	*P*. *strigosozonata* genome	*P*. *strigosozonata* transcriptome
Aspen	A	89,421,640	95.7	81.6
Aspen	B	121,720,712	98.6	83.5
Aspen + oil	A	45,205,066	93.9	80.8
Aspen + oil	B	316,975,014	91.6	78.8
Pine	A	72,936,232	99.4	85.6
Pine	B	62,199,846	99.2	86.4
Pine + oil	A	62,287,744	99.0	86.7
Pine + oil	B	64,422,628	99.2	86.9

Differential expression analyses in DESeq recovered 119 transcripts that were expressed ≥ log_2_(2-fold) across pairs of conditions with an adjusted p-value ≤ 0.01. Of these, 47 transcripts had predicted proteins without putative functions (S3 Table). Overall, the differentially-expressed transcripts accumulated to a greater extent in aspen media vs. pine media, and in the absence vs. presence of Bunker C oil. Specifically, transcripts accumulated in aspen without oil (40 upregulated) relative to pine without oil (21 upregulated), in aspen with oil (44 upregulated) relative to pine with oil (11 upregulated), in aspen without oil (15 upregulated) relative to aspen with oil (9 upregulated), and in pine without oil (13 upregulated) relative to pine with oil (5 upregulated) (S3 Table).

Of the nine transcripts that accumulated in aspen media with oil relative to aspen media without oil, only four had predicted protein domains with putative functions (Table 3). Two of these upregulated genes encoded WD40 repeats, which serve as scaffolds for multi-protein interactions (http://pfam.xfam.org/family/PF00400). The other two transcripts that were up-regulated in aspen media containing oil vs. aspen media without oil were oxidoreductases. One is a member of the 2-oxoglutarate and Fe(II)-dependent oxygenase superfamily, which in fungi has roles in synthesizing antibiotics like cephalosporin [42]. The other is a cytochrome p450 monooxygenase (CYP). CYPs oxidize a wide variety of substrates and have been implicated in degrading alkanes and PAHs [10, 12, 43, 44].

**Table 3:** *Punctularia strigosozonata* transcripts with predicted protein functions expressed ≥ log_2_(2-fold) (adjusted p < 0.01) in comparisons of 20-day growth on aspen and pine media with and without Bunker C oil.

	Log_2_ fold changes between media treatments				
Transcript ID	Aspen + oil Aspen	Pine + oil Pine	Pine Aspen	Pine + oil Aspen + oil	Predicted protein functions
	Carbohydrate Metabolic Processes				
136601				-4.78	CE16
134638	-3.43		-5.67		CE4
144208			-3.42		CBM13
144207				-4.86	CBM13
52241			-3.22	-5.32	GH10 with CBM1
55472			-3.20	-4.40	GH12 with CBM1
141097		-2.81		-3.59	GH13 with CBM 20
138371				-4.71	GH44
120145			-2.84	-4.50	GH45
112013				-3.96	GH5 with CBM1
71107				-5.10	GH7
76154				-4.59	GH7
79258			-3.17	-5.68	GH74 with CBM1
99251				-3.03	GH76
	Other Hydrolytic Activities				
109773		2.56			Carboxylesterase, type B
126655				-4.41	GH25
122284			-2.78		Poly(ADP-ribose) glycohydrolase
86290				2.68	Dienelactone hydrolase
	Oxidoreductase Activities				
65756	3.39				2OG-Fe(II) oxygenase
113634	-4.36		-8.24	-5.05	Aldo/keto reductase
88982			-2.83		Cytochrome c oxidase, subunit I
133687	4.15		4.59		CYP, B-class
139192	-5.59		-5.20		CYP, E-class, group I
55809			-2.43		CYP, E-class, group I
74484			-5.88		CYP, E-class, group IV
136122				-3.21	CYP, group I
135713			-5.66	-4.23	CYP, group IV
145671				-3.30	CYP, E-class, group I
53596				-4.23	Indoleamine 2,3-dioxygenase
107304				-3.52	Iron reductase domain/GMC oxidoreductase
75029				-3.46	Lytic polysaccharide monooxygenase
96689				-4.09	Lytic polysaccharide monooxygenase with CBM1
134993			-3.21	-6.95	Lytic polysaccharide monooxygenase with CBM1
116630			-3.05	-5.49	Lytic polysaccharide monooxygenase with CBM1
60310			4.08		Oxidoreductase, molybdopterin-binding
92239			-6.15	-4.76	Oxidoreductase; multicopper oxidase
141611			2.61		Short-chain dehydrogenase/reductase
55402			-8.43	-7.51	Zinc-binding alcohol dehydrogenase
	Cellular Functions				
144468		2.66			6-O-methylguanine DNA methyltransferase
58133		-3.11			AAA ATPase
106519				2.57	AAA ATPase, Mitochondrial chaperone BCS1
106411	-2.98		-5.58	-3.92	ABC transporter
55532			-3.40	-4.31	Acetyltransferase
74867			2.69		AMP-binding enzyme
107193			-3.58	-4.07	AMP-binding enzyme
63080			-2.30	-2.70	Aspartate protease
138654				-3.10	Aspartate protease
88642	-2.46				Ceratoplatanin
88726			-3.96		Ceratoplatanin
137633				3.76	F-box
21108			-2.63		Fungal hydrophobin
61618	-3.77	-3.85			Heat shock protein family 20
111941	-4.22	-3.63	-3.57	-2.98	Heat shock protein family 20
125088	-4.32	-3.67	-3.29		Heat shock protein family 20
142140		-3.79			Heat shock protein family 20
142147	-3.68	-4.62		-2.96	Heat shock protein family 20
105398		-2.61			Heat shock protein family 70
55838				-3.16	Major facilitator superfamily
93304			-2.53		Major facilitator superfamily
62703			-4.45		Major facilitator superfamily
67549			2.88		Major facilitator superfamily
100315				-2.98	Major facilitator superfamily
117799			-3.44		Major facilitator superfamily
114404		-4.40		-5.35	Peptidase A4 family
121226			-2.77		Per1-like
116001	-3.03				Polyketide cyclase
31014			3.49		Protein kinase
146483			3.23	3.83	Sodium/solute symporter family
134858			-2.86		Terpene synthase
118119		-4.11			Thaumatin-like protein
78026	3.51				WD40 repeat
145078	2.98				WD40 repeat
139219				-3.70	Zinc finger motif of a fungal transcription factor
77440			-3.10		Zinc finger domain

Positive log_2_ fold changes indicate transcript accumulation in the first treatment while negative log_2_ fold changes indicate transcript accumulation in the second treatment.

Of the five transcripts that accumulated in pine media with oil relative to pine media without oil, only two had predicted protein domains with putative functions (Table 3). One predicted gene model is a carboxylesterase, which has been shown to hydrolyze short-chain triglycerides [45]. The other is a DNA methyltransferase.

DESeq identified 14 transcripts with putative roles in carbohydrate metabolism that were differentially expressed ≥ log_2_(2-fold) (p ≤ 0.01) in condition comparisons (Table 3). Glycoside hydrolases (GH) catalyze the hydrolysis of glycosidic bonds between multiple carbohydrates or between carbohydrates and other molecules. Carbohydrate esterases (CE) catalyze the hydrolysis of esters; carbohydrate-specific esterases facilitate GH activities on complex carbohydrates with ester modifications [46]. Differences in GH and CE expression between cultures grown with and without oil in the media were minimal; one GH13 with a carbohydrate binding module (CBM) was downregulated in pine media with oil relative to plain pine media and one CE4 was downregulated in aspen media with oil relative to plain aspen media (Table 3). However, there were 14 gene models with putative roles in carbohydrate metabolism that were differentially expressed on pine and aspen media with and without oil. All 14 transcripts were downregulated on pine relative to aspen (Table 3).

This pattern of transcript accumulation in aspen media relative to pine media was observed in all of the other putative gene model function categories with the exception of the uncharacterized transcripts (S3 Table). Out of 19 oxidoreductase genes differentially expressed between cultures growing on aspen and pine media with and without oil, 16 were upregulated in aspen relative to pine (Table 3). Out of 26 gene models with various cellular maintenance functions differentially expressed on pine and aspen media, both with and without oil, 20 were upregulated in aspen relative to pine media. There were 17 uncharacterized transcripts differentially expressed in comparisons of pine and aspen media. Here, 8 transcripts accumulated in aspen media while the remaining 9 accumulated in pine media (S3 Table).

## Discussion

The primary objective of this study was to determine if white rot fungi are able to degrade Number 6 “Bunker C” fuel oil. The five dikaryotic fungal strains grown for 180 days in the presence of Bunker C oil reduced the amounts of phenanthrene, a C10 alkane, and a C14 alkane relative to the amounts of these compounds in undegraded Bunker C oil. The degradation of these compounds demonstrates the promise that white-rot fungi hold as agents of bioremediation for similar oil compounds. All cultures reduced the C10 alkane more than they reduced phenanthrene and the C14 alkane. Other studies with mixed and pure bacterial and fungal cultures have shown preferential degradation of short-chain alkanes, which may explain the difference in average degradation efficiency between the shorter C10 and longer C14 alkanes after 6 months (98.1% and 48.6%, respectively) [47–50].

Variation between species biological replicates in degradation of the C14 alkane and phenanthrene was evident for three of the four species with biological replicates on the same spawn substrate. It is likely that there is variation in degradation ability that was not captured by culturing only one or two replicates per species. Further, as *P*. *strigosozonata* was replicated only at 20 days on pine and aspen spawn but not at 180 days, it is uncertain whether the inability of the *P*. *strigosozonata* monokaryon to degrade the C14 alkane and phenanthrene in pine and aspen media is attributable to the short time span of culturing or to real species-specific differences between this strain and the other five that were grown for 180 days. This result is in contrast to another study of phenanthrene degradation with a white-rot fungus, *P*. *ostreatus*, which showed a reduction of the PAH by 94% in defined media after only 11 days [51]. Plant extractive compounds in the wood sawdust-based growth media used in this study could have interfered with rapid degradation of phenanthrene and other complex compounds in Bunker C oil.

The secondary objective of this study was to assess whether there are differences in gene expression by *P*. *strigosozonata* after 20 days of growth on pine and aspen sawdust media and with or without Bunker C oil. Of the 14 transcripts that were upregulated in oil-containing media, only 6 had putative functional characterizations. Three of these encoded putative oxidoreductases, suggesting that oxidative enzymes were partially responsible for the oil degradation observed in *P*. *strigosozonata* cultures. Since the *P*. *strigosozonata* cultures did not appreciably degrade phenanthrene or the C14 alkane, we conclude that the differentially expressed transcripts may have been involved in short-chain alkane biotransformation. CYP enzymes, like the one upregulated in aspen media containing Bunker C oil relative to plain aspen media, catalyze terminal oxidation of alkanes [12] and are involved in early stages of PAH biotransformation [9, 10, 44]. The upregulated CYP in aspen media containing oil could have been responsible for the small amount (5.1%) of phenanthrene transformation observed in that treatment. However, this pattern of upregulation in oil-containing media did not hold for the other six differentially expressed genes identified as P450s in this study, which were upregulated in aspen media relative to pine media. Carboxylesterases, like the one upregulated in pine media containing Bunker C oil relative to plain pine media, are involved in phase I metabolism of xenobiotics in diverse organisms [52]. Further, one study demonstrated increased activity of a *Fusarium* carboxylesterase when olive oil was added to the growth medium [53]. The genes involved in early phases of xenobiotic biotransformation that were upregulated in cultures containing Bunker C oil may be induced detoxification responses. In addition, there were 8 transcripts without functional characterizations that accumulated in media with oil relative to media without oil (S3 Table). These genes could have important but as-yet undiscovered roles in biotransformation of crude oil.

Fourteen transcripts with putative proteins related to carbohydrate metabolism were differentially expressed in one or more condition comparisons. All of these genes were upregulated in aspen media relative to pine media. The extent of overexpression may indicate that *P*. *strigosozonata* was metabolizing carbohydrates more actively in aspen spawn than in pine spawn at the time the cultures were frozen at -80°C to prevent further growth. Ginns and Lefebvre [30] list *Picea sp*. as one of 34 hosts for *P*. *strigosozonata*, but most reports are on hardwoods, including eight species of *Populus* (aspen). We speculate that the enhanced expression of diverse genes related to carbohydrate metabolism on aspen vs. pine by *P*. *strigosozonata* may reflect adaption to hardwood substrates, specifically *Populus* [30]. Vanden Wymelenberg *et al*. [54] demonstrated upregulated transcript levels in the white-rot fungus *P*. *chrysosporium* in aspen (*Populus grandidentata)* media relative to pine (*Pinus strobus*) media. In parallel with the results from the study detailed in this paper, many of the upregulated genes were for GH enzymes with putative roles in carbohydrate metabolism.

*Irpex lacteus*, *T*. *biforme*, *P*. *radiata*, *T*. *versicolor*, and *P*. *ostreatus* are promising candidates for degrading petroleum compounds, but the mechanisms of Bunker C oil degradation remain obscure. The hydrocarbon degradation screen completed in this study should be replicated in the bioremediation system at Fisherville Mill to assess the effects of lower ambient temperatures, lack of control over media sterility and nutrient content, and the flow rate of polluted canal water through fungal spawn. The results of the degradation analysis of the 20-day *P*. *strigosozonata* cultures suggest that white-rot fungi are able to rapidly degrade short *n*-alkanes, but continuous monitoring of filtered canal water would help determine if white-rot fungi can appreciably degrade phenanthrene and long chain *n-*alkanes in fewer than 180 days. Comparative gene expression analyses revealed greater differences between expression profiles of *P*. *strigosozonata* grown on different woody substrates than between profiles of this fungus growing in the presence and absence of Bunker C oil, suggesting that oil biotransformation may be less of an induced mechanism than a co-oxidative process that occurs alongside lignocellulose metabolic processes.

## Supporting Information

S1 Fig Flame ionization detector chromatogram of terpene (hopane) in control samples. In labeled terpene peak, top line: autoclaved Bunker C oil, middle line: uninoculated pine spawn without oil, bottom line: uninoculated aspen spawn without oil.
(JPG)

S2 Fig Flame ionization detector chromatogram of phenanthrene in control samples. In labeled phenanthrene peak, top line: autoclaved Bunker C oil, middle line: uninoculated pine spawn without oil, bottom line: uninoculated aspen spawn without oil.
(JPG)

S3 Fig Flame ionization detector chromatogram of C10 and C14 alkanes in control samples. In labeled peaks, top line: Bunker C oil, middle line: uninoculated pine spawn without oil, bottom line: uninoculated aspen spawn without oil.
(JPG)

S4 Fig Dispersion plot of normalized RNA-Seq read counts from all *Punctularia strigosozonata* samples. (JPG)

S1 Table Quantity (μg) and quality (absorbance ratios) of LiCl-purified total *Punctularia strigosozonata* RNA. (DOCX)

S2 Table Pearson correlations (r) between normalized gene counts of *Punctularia strigosozonata* biological replicate RNA-Seq libraries. (DOCX)

S3 Table *Punctularia strigosozonata* transcripts differentially expressed ≥ log_2_(2-fold) (adjusted p ≤ 0.01) in condition comparisons identified using R package DESeq. Positive log_2_ fold changes indicate accumulation of transcripts in the first condition while negative changes indicate accumulation of transcripts in the second condition. Putative transcript functions were characterized using the JGI Mycocosm database and Pfam.
(DOCX)

S1 Dataset Counts table of transcripts that mapped to the *Punctularia strigosozonata* genome across all culture conditions. (TXT)

## References

[1] Boddy L. Development and function of fungal communities in decomposing wood. Mycology Series. 1992;9:749–82.

[2] Gadd GM. Fungi and industrial pollutants In: Kubicek CP, Druzhinina IS, editors. Environmental and Microbial Relationships. The Mycota. IV 2nd ed. Berlin Heidelberg: Springer-Verlag; 2007 p. 69–84.

[3] Floudas D, Binder M, Riley R, Barry K, Blanchette RA, Henrissat B, et al The Paleozoic origin of enzymatic lignin decomposition reconstructed from 31 fungal genomes. Science. 2012;336(6089):1715–9. 10.1126/science.1221748 22745431

[4] Kanaly R, Hur H. Growth of *Phanerochaete chrysosporium* on diesel fuel hydrocarbons at neutral pH. Chemosphere. 2006;63(2):202–11. 16226785 10.1016/j.chemosphere.2005.08.022

[5] Harvey PJ, Thurston CF. The biochemistry of ligninolytic fungi In: Gadd GM, editor. Fungi in Bioremediation. Cambridge: Cambridge University Press; 2001 p. 27–51.

[6] Martinez AT. Molecular biology and structure-function of lignin-degrading heme peroxidases. Enzyme Microb Technol. 2002;30(4):425–44.

[7] Baldrian P. Fungal laccases: occurrence and properties. FEMS Microbiol Rev. 2006;30(2):215–42. 16472305 10.1111/j.1574-4976.2005.00010.x

[8] Asgher M, Bhatti HN, Ashraf M, Legge RL. Recent developments in biodegradation of industrial pollutants by white rot fungi and their enzyme system. Biodegradation. 2008;19(6):771–83. 10.1007/s10532-008-9185-3 18373237

[9] Bamforth SM, Singleton I. Bioremediation of polycyclic aromatic hydrocarbons: current knowledge and future directions. J Chem Technol Biotechnol. 2005;80(7):723–36.

[10] Syed K, Porollo A, Lam YW, Grimmett PE, Yadav JS. CYP63A2, a catalytically versatile fungal P450 monooxygenase capable of oxidizing higher-molecular-weight polycyclic aromatic hydrocarbons, alkylphenols, and alkanes. Appl Environ Microbiol. 2013;79(8):2692–702. 10.1128/aem.03767-12 23416995 PMC3623170

[11] Moen MA, Hammel KE. Lipid peroxidation by the manganese peroxidase of *Phanerochaete chrysosporium* is the basis for phenanthrene oxidation by the intact fungus. Appl Environ Microbiol. 1994;60(6):1956–61. 16349285 10.1128/aem.60.6.1956-1961.1994 PMC201586

[12] Van Beilen JB, Li Z, Duetz WA, Smits TH, Witholt B. Diversity of alkane hydroxylase systems in the environment. Oil Gas Sci Technol. 2003;58(4):427–40.

[13] Cajthaml T, Erbanová P, Kollmann A, Novotný Č, Šašek V, Mougin C. Degradation of PAHs by ligninolytic enzymes of *Irpex lacteus* . Folia Microbiol (Praha). 2008;53(4):289–94. 18759111 10.1007/s12223-008-0045-7

[14] Kim HY, Song HG. Transformation and mineralization of 2, 4, 6-trinitrotoluene by the white rot fungus *Irpex lacteus* . Appl Microbiol Biotechnol. 2003;61(2):150–6. 12655457 10.1007/s00253-002-1211-5

[15] Svobodová K, Majcherczyk A, Novotnı C, Kües U. Implication of mycelium-associated laccase from Irpex lacteus in the decolorization of synthetic dyes. Bioresour Technol. 2008;99(3):463–71. 17369037 10.1016/j.biortech.2007.01.019

[16] Shin K, Kim YH, Lim J. Purification and characterization of manganese peroxidase of the white-rot fungus *Irpex lacteus* . J Microbiol. 2005;43(6):503–9. 16410766

[17] Novotny C, Cajthaml T, Svobodova K, Susla M, Sasek V. *Irpex lacteus*, a white-rot fungus with biotechnological potential—review. Folia Microbiol (Praha). 2009;54(5):375–90. Epub 2009/11/26. PubMed . 19937209 10.1007/s12223-009-0053-2

[18] Novotny C, Erbanova P, Cajthaml T, Rothschild N, Dosoretz C, Sasek V. *Irpex lacteus*, a white rot fungus applicable to water and soil bioremediation. Appl Microbiol Biotechnol. 2000;54(6):850–3. Epub 2001/01/11. PubMed . 11152080 10.1007/s002530000432

[19] Bezalel L, Hadar Y, Cerniglia CE. Mineralization of polycyclic aromatic hydrocarbons by the white rot fungus *Pleurotus ostreatus* . Appl Environ Microbiol. 1996;62(1):292–5. 16535219 10.1128/aem.62.1.292-295.1996 PMC1388760

[20] Pozdnyakova NN, Nikitina VE, Turovskaya OV. Bioremediation of oil-polluted soil with an association including the fungus *Pleurotus ostreatus* and soil microflora. Appl Biochem Microbiol. 2008;44(1):60–5. 18491600

[21] Aggelis G, Iconomou D, Christou M, Bokas D, Kotzailias S, Christou G, et al Phenolic removal in a model olive oil mill wastewater using *Pleurotus ostreatus* in bioreactor cultures and biological evaluation of the process. Water Res. 2003;37(16):3897–904. 12909108 10.1016/S0043-1354(03)00313-0

[22] Collins PJ, Kotterman M, Field JA, Dobson A. Oxidation of anthracene and benzo[a]pyrene by laccases from *Trametes versicolor* . Appl Environ Microbiol. 1996;62(12):4563–7. 16535468 10.1128/aem.62.12.4563-4567.1996 PMC1389006

[23] Field JA, de Jong E, Feijoo Costa G, de Bont JA. Biodegradation of polycyclic aromatic hydrocarbons by new isolates of white rot fungi. Appl Environ Microbiol. 1992;58(7):2219–26. Epub 1992/07/01. PubMed 1637159 10.1128/aem.58.7.2219-2226.1992 PMC195758

[24] Novotny C, Erbanova P, Sasek V, Kubatova A, Cajthaml T, Lang E, et al Extracellular oxidative enzyme production and PAH removal in soil by exploratory mycelium of white rot fungi. Biodegradation. 1999;10(3):159–68. Epub 1999/09/24. PubMed . 10492884 10.1023/a:1008324111558

[25] Rogalski J, Lundell TK, Leonowicz A, Hatakka AI. Influence of aromatic compounds and lignin on production of ligninolytic enzymes by *Phlebia radiata* . Phytochemistry. 1991;30(9):2869–72.

[26] Eshghi H, Alishahi Z, Zokaei M, Daroodi A, Tabasi E. Decolorization of methylene blue by new fungus: *Trichaptum biforme* and decolorization of three synthetic dyes by *Trametes hirsuta* and *Trametes gibbosa* . Eur J Chem. 2011;2(4):463–8.

[27] Freitag M, Morrell JJ. Decolorization of the polymeric dye Poly R-478 by wood-inhabiting fungi. Can J Microbiol. 1992;38(8):811–22.

[28] Farr D, Rossman A. Fungal Databases. Systematic Mycology and Microbiology Laboratory, ARS, USDA 2013.

[29] Gilbertson RL, Ryvarden L. North American Polypores Oslo: Fungiflora; 1987.

[30] Ginns J, Lefebvre M. Lignicolous corticioid fungi (Basidiomycota) of North America: systematics, distribution, and ecology Saint Paul: American Phytopathological Society; 1993.

[31] Ollila P, Soukup J, Nowack B, Bernat E, Brammer D, Hultstrom E, et al., editors. Fisherville Mill–a case study–cost effective remediation through collaboration. Proceedings of the Annual International Conference on Soils, Sediments, Water and Energy; 2008: Berkeley Electronic Press.

[32] Kristanti RA. Bioremediation of Crude Oil by White Rot Fungi Polyporus sp. S133. Journal of Microbiology and Biotechnology. 2011;21(9):995–1000. 10.4014/jmb.1105.05047 21952378

[33] Marquez-Rocha FJ, Hernandez-Rodrıguez VZ, Vazquez-Duhalt R. Biodegradation of soil-adsorbed polycyclic aromatic hydrocarbons by the white rot fungus Pleurotus ostreatus. Biotechnology Letters. 2000;22:469–72.

[34] U.S. Environmental Protection Agency. Method 8015C Nonhalogenated Organics by Gas Chromatography 2007.

[35] Wang Z, Fingas M, Owens E, Sigouin L, Brown C. Long-term fate and persistence of the spilled Metula oil in a marine salt marsh environment: Degradation of petroleum biomarkers. Journal of Chromatography A. 2001;926(2):275–90. 11556333 10.1016/s0021-9673(01)01051-2

[36] Wang J, Zhang X, Li G. Compositions and diagnostic ratios of heavily degraded crude oil residues in contaminated soil in oilfields. Huan Jing Ke Xue. 2012;33(4):1352–60. 22720589

[37] da Cruz GF, dos Santos Neto EV, Marsaioli AJ. Petroleum degradation by aerobic microbiota from the Pampo Sul oil field, Campos Basin, Brazil. Organic Geochemistry. 2008;39(8):1204–9.

[38] Li H, Durbin R. Fast and accurate long-read alignment with Burrows–Wheeler transform. Bioinformatics. 2010;26(5):589–95. 10.1093/bioinformatics/btp698 20080505 PMC2828108

[39] Anders S, Huber W. Differential expression analysis for sequence count data. Genome Biol. 2010;11(10):R106 10.1186/gb-2010-11-10-r106 20979621 PMC3218662

[40] Grigoriev I, Nikitin R, Haridas S, Kuo A, Ohm R, Otillar R, et al Mycocosm portal: gearing up for 1000 fungal genomes. Nucleic Acids Research. 2014;42(1):D699–704. 24297253 10.1093/nar/gkt1183 PMC3965089

[41] Finn RD, Bateman A, Clements J, Coggill P, Eberhardt RY, Eddy SR, et al The Pfam protein families database. Nucleic Acids Research. 2014;42(D1):D222–D30. 24288371 10.1093/nar/gkt1223 PMC3965110

[42] Valegård K, Terwisscha van Scheltinga A, Lloyd M, Hara T, Ramaswamy S, Perrakis A, et al Structure of a cephalosporin synthase. Nature. 1998;394:805–9. 9723623 10.1038/29575

[43] van Beilen JB, Funhoff EG, van Loon A, Just A, Kaysser L, Bouza M, et al Cytochrome P450 Alkane Hydroxylases of the CYP153 Family Are Common in Alkane-Degrading Eubacteria Lacking Integral Membrane Alkane Hydroxylases. Appl Environ Microbiol. 2006;72(1):59–65. 10.1128/aem.72.1.59-65.2006 16391025 PMC1352210

[44] Syed K, Doddapaneni H, Subramanian V, Lam YW, Yadav JS. Genome-to-function characterization of novel fungal P450 monooxygenases oxidizing polycyclic aromatic hydrocarbons (PAHs). Biochemical and Biophysical Research Communications. 2010;399(4):492–7. 10.1016/j.bbrc.2010.07.094 20674550 PMC2943217

[45] Bornscheuer U. Microbial carboxyl esterases: classification, properties and application in biocatalysis. FEMS Microbiol Rev. 2002;26:73–81. 12007643 10.1111/j.1574-6976.2002.tb00599.x

[46] Cantarel BL, Coutinho PM, Rancurel C, Bernard T, Lombard V, Henrissat B. The Carbohydrate-Active EnZymes database (CAZy): an expert resource for Glycogenomics. Nucleic Acids Research. 2009;37(suppl 1):D233–D8. 18838391 10.1093/nar/gkn663 PMC2686590

[47] Atlas RM. Microbial degradation of petroleum hydrocarbons: an environmental perspective. Microbiol Rev. 1981;45(1):180–209. 7012571 10.1128/mr.45.1.180-209.1981 PMC281502

[48] Zajic J, Supplisson B. Emulsification and degradation of “Bunker C” fuel oil by microorganisms. Biotechnol Bioeng. 1972;14(3):331–43. 5029879 10.1002/bit.260140306

[49] Mulkins-Phillips G, Stewart JE. Effect of environmental parameters on bacterial degradation of bunker C oil, crude oils, and hydrocarbons. Appl Microbiol. 1974;28(6):915–22. 4451374 10.1128/am.28.6.915-922.1974 PMC186856

[50] Vandermeulen J, Singh J. Arrow oil spill, 1970–90: Persistence of 20-yr weathered bunker C fuel oil. Can J Fish Aquat Sci. 1994;51(4):845–55.

[51] Bezalel L, Hadar Y, Fu PP, Freeman JP, Cerniglia CE. Metabolism of phenanthrene by the white rot fungus *Pleurotus ostreatus* . Appl Environ Microbiol. 1996;62(7):2547–53. 8779594 10.1128/aem.62.7.2547-2553.1996 PMC168037

[52] Parkinson A. Biotransformation of xenobiotics In: Klaassen C, editor. Casarett and Doull's Toxicology: The Basic Science of Poisons. New York: McGraw-Hill; 2001 p. 133–224.

[53] Donaghy J, McKay AM. Extracellular carboxylesterase activity of *Fusarium graminearum* . Appl Microbiol Biotechnol. 1992;37:742–4.

[54] Wymelenberg AV, Gaskell J, Mozuch M, Splinter S, Durant B, Sabat G, et al Significant Alteration of Gene Expression in Wood Decay Fungi *Postia placenta* and *Phanerochaete chrysosporium* by Plant Species. Appl Environ Microbiol. 2011;77(13):4499–507. 10.1128/AEM.00508-11 21551287 PMC3127733

