# Supplementary figures and images for: Degradation of Bunker C Fuel Oil by White-Rot Fungi in Sawdust Cultures Suggests Potential Applications in Bioremediation
Source: PLoS One. 2015 Jun 25;10(6):e0130381. doi: 10.1371/journal.pone.0130381 (PMC4482389; doi:10.1371/journal.pone.0130381)

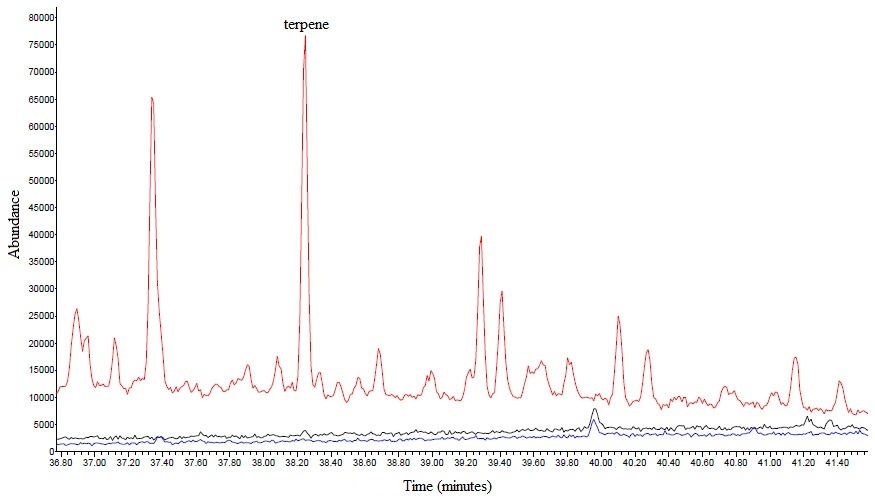

Supplement: S1 Fig — In labeled terpene peak, top line: autoclaved Bunker C oil, middle line: uninoculated pine spawn without oil, bottom line: uninoculated aspen spawn without oil. (JPG) [file pone.0130381.s001.jpg]

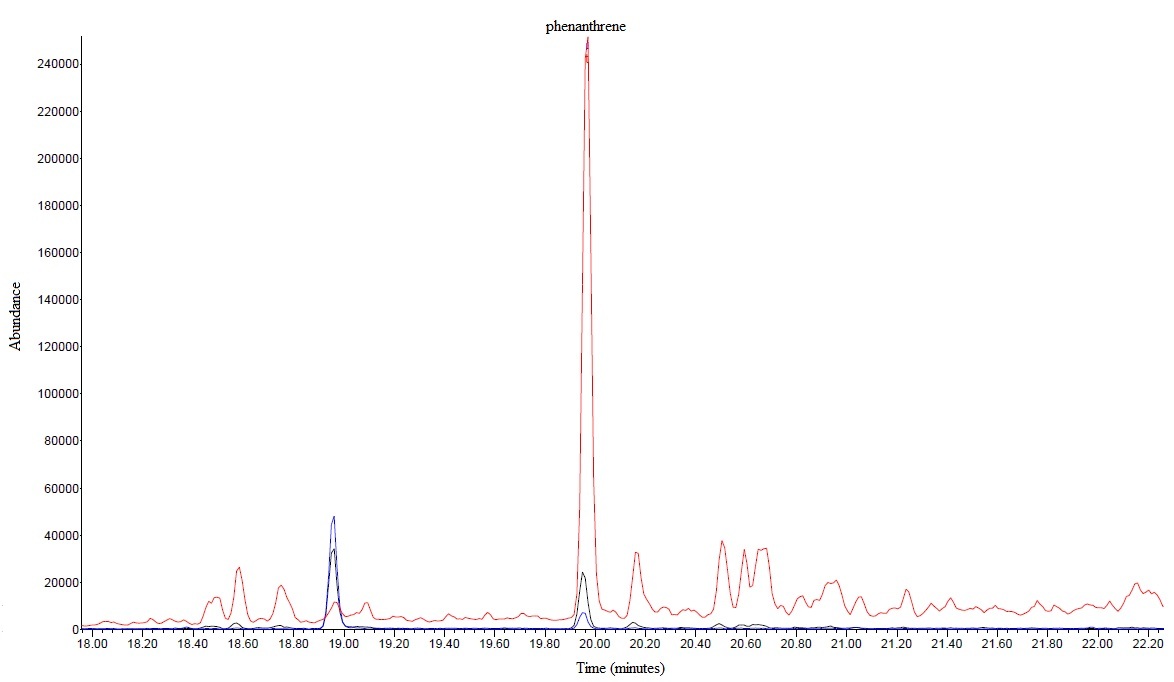

Supplement: S2 Fig — In labeled phenanthrene peak, top line: autoclaved Bunker C oil, middle line: uninoculated pine spawn without oil, bottom line: uninoculated aspen spawn without oil. (JPG) [file pone.0130381.s002.jpg]

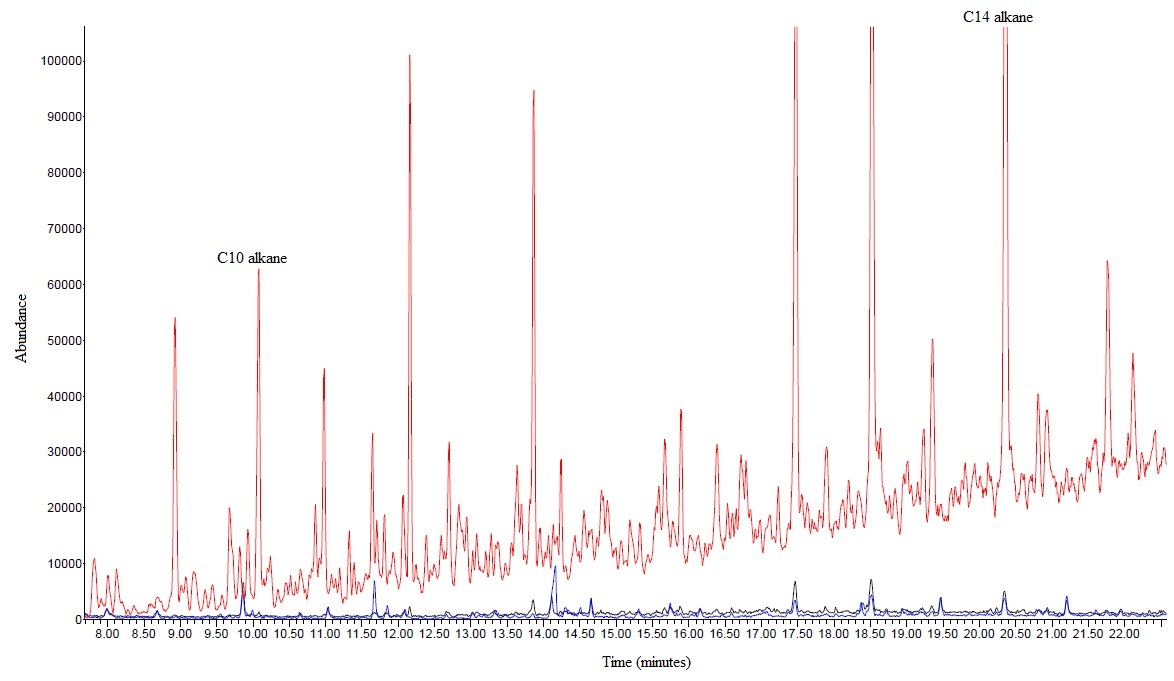

Supplement: S3 Fig — In labeled peaks, top line: Bunker C oil, middle line: uninoculated pine spawn without oil, bottom line: uninoculated aspen spawn without oil. (JPG) [file pone.0130381.s003.jpg]

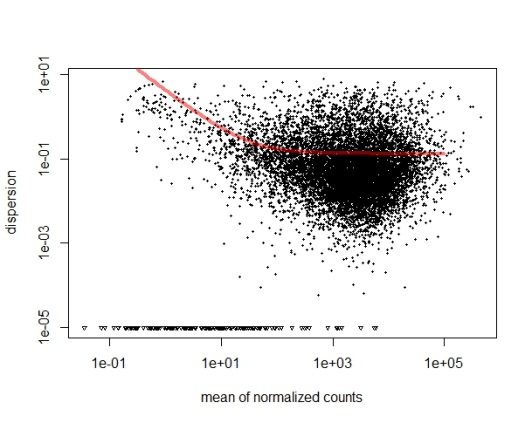

Supplement: S4 Fig — (JPG) [file pone.0130381.s004.jpg]
