# Supplementary material for: Degradation of Bunker C Fuel Oil by White-Rot Fungi in Sawdust Cultures Suggests Potential Applications in Bioremediation
Source: PLoS One. 2015 Jun 25;10(6):e0130381. doi: 10.1371/journal.pone.0130381 (PMC4482389; doi:10.1371/journal.pone.0130381)
Supplement: S1 Table — (DOCX) [file pone.0130381.s005.docx]

| TABLE S1. Quantity (µg) and quality (absorbance ratios) of LiCl-purified total *Punctularia strigosozonata total* RNA. | | | | | |
| --- | --- | --- | --- | --- | --- |
|  | |  |  |  |  |
| Sample | Replicate | Mass (µg) | Purity (A_260_/A_280_) | Purity (A_260_/A_230_) | Concentration (ng/µL) |
| Aspen | A | 38.2 | 2.1 | 1.88 | 332.4 |
| Aspen | B | 38.5 | 2.17 | 2.01 | 366.8 |
| Aspen + oil | A | 16.6 | 2.19 | 1.84 | 237.0 |
| Aspen + oil | B | 40.6 | 2.09 | 1.89 | 368.9 |
| Pine | A | 10.1 | 0.98 | 2.68 | 281.4 |
| Pine | B | 29.6 | 1.05 | 0.96 | 388.9 |
| Pine & oil | A | 49.5 | 1.27 | 2.37 | 317.0 |
| Pine + oil | B | 32.0 | 1.06 | 0.96 | 450.2 |
