# Supplementary material for: Degradation of Bunker C Fuel Oil by White-Rot Fungi in Sawdust Cultures Suggests Potential Applications in Bioremediation
Source: PLoS One. 2015 Jun 25;10(6):e0130381. doi: 10.1371/journal.pone.0130381 (PMC4482389; doi:10.1371/journal.pone.0130381)
Supplement: S2 Table — (DOCX) [file pone.0130381.s006.docx]

| TABLE S2. Pearson correlations (r) between normalized gene counts of *Punctularia strigosozonata* biological replicate RNA-Seq libraries. | | | | | | | | | |
| --- | --- | --- | --- | --- | --- | --- | --- | --- | --- |
| Media treatment |  | Aspen | | Pine + oil | | Pine | | Aspen + oil | |
| Replicate |  | B | A | A | B | A | B | A | B |
| Aspen | B | ~ | 0.76 | 0.63 | 0.60 | 0.82 | 0.71 | 0.35 | 0.66 |
| Aspen | A | 0.76 | ~ | 0.61 | 0.57 | 0.67 | 0.70 | 0.62 | 0.74 |
| Pine + oil | A | 0.63 | 0.61 | ~ | 0.98 | 0.75 | 0.91 | 0.37 | 0.65 |
| Pine + oil | B | 0.60 | 0.57 | 0.98 | ~ | 0.73 | 0.88 | 0.34 | 0.61 |
| Pine | A | 0.82 | 0.67 | 0.75 | 0.73 | ~ | 0.85 | 0.36 | 0.61 |
| Pine | B | 0.71 | 0.70 | 0.91 | 0.88 | 0.85 | ~ | 0.41 | 0.67 |
| Aspen + oil | A | 0.35 | 0.62 | 0.37 | 0.34 | 0.36 | 0.41 | ~ | 0.73 |
| Aspen + oil | B | 0.66 | 0.74 | 0.65 | 0.61 | 0.61 | 0.67 | 0.73 | ~ |
