# Supplementary material for: Degradation of Bunker C Fuel Oil by White-Rot Fungi in Sawdust Cultures Suggests Potential Applications in Bioremediation
Source: PLoS One. 2015 Jun 25;10(6):e0130381. doi: 10.1371/journal.pone.0130381 (PMC4482389; doi:10.1371/journal.pone.0130381)
Supplement: S3 Table — Positive log2 fold changes indicate accumulation of transcripts in the first condition while negative changes indicate accumulation of transcripts in the second condition. Putative transcript functions were characterized using the JGI Mycocosm database and Pfam. (DOCX) [file pone.0130381.s007.docx]

| Table S3. *Punctularia strigosozonata* transcripts expressed ≥2-fold (adjusted p < 0.01) in comparisons of 20-day growth on aspen and pine media with and without Bunker C oil. Positive log_2_ fold changes indicate transcript accumulation in the first treatment while negative log_2_ fold changes indicate transcript accumulation in the second treatment. | | | | | | | |
| --- | --- | --- | --- | --- | --- | --- | --- |
|  |  | Log_2_ fold changes between media treatments | | | |  |  |
| Transcript  ID | Protein  ID | Aspen + oil Aspen | Pine + oil Pine | Pine Aspen | Pine + oil Aspen + oil | Pfam domains | Putative function |
|  | Carbohydrate Metabolic Processes | | | |  |  |  |
| 136601 | 136495 |  |  |  | -4.777 | PF00657 | CE16 |
| 134638 | 134532 | -3.433 |  | -5.668 |  | PF01522 | CE4 |
| 144208 | 144102 |  |  | -3.424 |  | PF00652 | CBM13 |
| 144207 | 144101 |  |  |  | -4.857 | PF00652 | CBM13 |
| 52241 | 52135 |  |  | -3.222 | -5.323 | PF00331 PF00734 | GH10 with CBM1 |
| 55472 | 55366 |  |  | -3.203 | -4.404 | PF00734 PF01670 | GH12 with CBM1 |
| 141097 | 140991 |  | -2.806 |  | -3.593 | PF00128 PF00686 | GH13 with CBM 20 |
| 138371 | 138265 |  |  |  | -4.715 | PS00599 | GH44 |
| 120145 | 120039 |  |  | -2.837 | -4.504 | NA | GH45 |
| 112013 | 111907 |  |  |  | -3.959 | PF00150 PF00734 | GH5 with CBM1 |
| 71107 | 71001 |  |  |  | -5.100 | PF00840 | GH7 |
| 76154 | 76048 |  |  |  | -4.586 | PF00840 | GH7 |
| 79258 | 79152 |  |  | -3.167 | -5.678 | PF00734 PF02012 | GH74 with CBM1 |
| 99251 | 99145 |  |  |  | -3.030 | PF03663 | GH76 |
|  | Other hydrolytic activities | | |  |  |  |  |
| 109773 | 109667 |  | 2.559 |  |  | PF00135 | carboxylesterase, type B |
| 126655 | 126549 |  |  |  | -4.415 | PF01183 | GH25 |
| 122284 | 122178 |  |  | -2.783 |  | PF05028 | glycohydrolase |
| 86290 | 86184 |  |  |  | 2.675 | PF01738 | hydrolase |
|  | Oxidoreductases | |  |  |  |  |  |
| 65756 | 65650 | 3.391 |  |  |  | PF03171 | 2OG-Fe(II) oxygenase |
| 113634 | 113528 | -4.364 |  | -8.240 | -5.054 | PF00248 | aldo/keto reductase |
| 88982 | 88876 |  |  | -2.834 |  |  | cytochrome c oxidase, subunit I |
| 133687 | 133581 | 4.152 |  | 4.587 |  | PF00067 | CYP, B-class |
| 139192 | 139086 | -5.590 |  | -5.204 |  | PF00067 | CYP, E-class, group I |
| 55809 | 55703 |  |  | -2.426 |  | PF00067 | CYP, E-class, group I |
| 74484 | 74378 |  |  | -5.880 |  | PF00067 | CYP, E-class, group IV |
| 136122 | 136016 |  |  |  | -3.213 | PF00067 | CYP, group I |
| 135713 | 135607 |  |  | -5.656 | -4.227 | PF00067 | CYP, group IV |
| 145671 | 145565 |  |  |  | -3.301 | PF00067 | CYP, E-class, group I |
| 53596 | 53490 |  |  |  | -4.225 | PF01231 | indoleamine 2,3-dioxygenase |
| 107304 | 107198 |  |  |  | -3.523 | PF01266 PF05199 | Iron reductase domain/GMC oxidoreductase |
| 75029 | 74923 |  |  |  | -3.457 | PF03443 | Lytic polysaccharide monooxygenase |
| 96689 | 96583 |  |  |  | -4.088 | PF00734 PF03443 | Lytic polysaccharide monooxygenase with CBM1 |
| 134993 | 134887 |  |  | -3.209 | -6.954 | PF00734 PF03443 | Lytic polysaccharide monooxygenase with CBM1 |
| 116630 | 116524 |  |  | -3.048 | -5.493 | PF03443 | Lytic polysaccharide monooxygenase with CBM1 |
| 60310 | 60204 |  |  | 4.083 |  | PF00174 PF03404 | Oxidoreductase, molybdopterin-binding |
| 92239 | 92133 |  |  | -6.154 | -4.755 | PF00394 PF07731 PF07732 | oxidoreductase; multicopper oxidase |
| 141611 | 141505 |  |  | 2.611 |  | PF00106 | short-chain dehydrogenase/reductase |
| 55402 | 55296 |  |  | -8.425 | -7.510 | PF00107 | zinc-binding alcohol dehydrogenase |
|  | Cellular Functions | |  |  |  |  |  |
| 144468 | 144362 |  | 2.658 |  |  | PF01035 | 6-O-methylguanine DNA methyltransferase |
| 58133 | 58027 |  | -3.111 |  |  | PF00004 | AAA ATPase |
| 106519 | 106413 |  |  |  | 2.570 | PF00004 PF08740 | AAA ATPase, Mitochondrial chaperone BCS1 |
| 106411 | 106305 | -2.977 |  | -5.576 | -3.919 | PF00005 | ABC transporter |
| 55532 | 55426 |  |  | -3.398 | -4.309 | PF00583 | Acetyltransferase |
| 74867 | 74761 |  |  | 2.689 |  | PF00501 | AMP-binding enzyme |
| 107193 | 107087 |  |  | -3.583 | -4.067 | PF00501 PF00550 | AMP-binding enzyme |
| 63080 | 62974 |  |  | -2.297 | -2.704 | PF00026 | Aspartate protease |
| 138654 | 138548 |  |  |  | -3.097 | PF00026 | Aspartate protease |
| 88642 | 88536 | -2.459 |  |  |  | PF07249 | ceratoplatanin |
| 88726 | 88620 |  |  | -3.964 |  | PF07249 | ceratoplatanin |
| 137633 | 137527 |  |  |  | 3.761 | PF00646 | F-box |
| 21108 | 21002 |  |  | -2.626 |  | PF01185 | fungal hydrophobin |
| 61618 | 61512 | -3.770 | -3.849 |  |  | PF00011 | Heat shock protein family 20 |
| 111941 | 111835 | -4.219 | -3.631 | -3.567 | -2.979 | PF00011 | Heat shock protein family 20 |
| 125088 | 124982 | -4.324 | -3.672 | -3.287 |  | PF00011 | Heat shock protein family 20 |
| 142140 | 142034 |  | -3.786 |  |  | PF00011 | Heat shock protein family 20 |
| 142147 | 142041 | -3.678 | -4.621 |  | -2.961 | PF00011 | Heat shock protein family 20 |
| 105398 | 105292 |  | -2.612 |  |  | PF00012 | Heat shock protein family 70 |
| 55838 | 55732 |  |  |  | -3.161 | PF00083 | major facilitator superfamily |
| 93304 | 93198 |  |  | -2.525 |  | PF00083 | Major facilitator superfamily |
| 62703 | 62597 |  |  | -4.447 |  | PF07690 | major facilitator superfamily |
| 67549 | 67443 |  |  | 2.884 |  | PF07690 | major facilitator superfamily |
| 100315 | 100209 |  |  |  | -2.983 | PF07690 | major facilitator superfamily |
| 117799 | 117693 |  |  | -3.442 |  | PF07690 | major facilitator superfamily |
| 114404 | 114298 |  | -4.399 |  | -5.346 | PF01828 | Peptidase A4 family |
| 121226 | 121120 |  |  | -2.774 |  | PF04080 | Per1-like |
| 116001 | 115895 | -3.027 |  |  |  | PF03364 | Polyketide cyclase |
| 31014 | 30908 |  |  | 3.488 |  | PF00069 | protein kinase |
| 146483 | 146377 |  |  | 3.227 | 3.827 | PF00474 | Sodium/solute symporter family |
| 134858 | 134752 |  |  | -2.857 |  | PF03936 | terpene synthase |
| 118119 | 118013 |  | -4.113 |  |  | PF00314 | thaumatin |
| 78026 | 77920 | 3.508 |  |  |  | PF00400 | WD40 repeat |
| 145078 | 144972 | 2.984 |  |  |  | PF00400 | WD40 repeat |
| 139219 | 139113 |  |  |  | -3.703 | PF00172 PF04082 | Zinc finger motif, fungal transcription factor |
| 77440 | 77334 |  |  | -3.102 |  | PF00097 | Zinc finger protein |
|  | Uncharacterized | |  |  |  |  |  |
| 46873 | 46767 | 4.133 |  |  |  |  |  |
| 47332 | 47226 |  |  | -2.531 |  |  |  |
| 48508 | 48402 |  |  |  | -4.784 |  |  |
| 54901 | 54795 |  | 2.726 |  |  |  |  |
| 56319 | 56213 |  |  | -3.539 | -3.525 |  |  |
| 74924 | 74818 |  |  |  | -3.904 |  |  |
| 75788 | 75682 |  |  |  | 3.139 |  |  |
| 77291 | 77185 |  |  |  | -3.987 |  |  |
| 108670 | 108564 |  |  | 2.974 |  |  |  |
| 118817 | 118711 |  | 2.642 |  |  |  |  |
| 119157 | 119051 |  |  |  | -3.218 |  |  |
| 126126 | 126020 | -4.000 |  | -4.939 |  |  |  |
| 126701 | 126595 | -5.830 |  |  |  |  |  |
| 128674 | 128568 | 3.767 |  | 2.680 |  |  |  |
| 130353 | 130247 |  |  | 2.427 |  |  |  |
| 131724 | 131618 |  |  | 2.795 |  |  |  |
| 139086 | 138980 |  |  | 3.078 | 3.540 |  |  |
| 143849 | 143743 |  |  |  | 4.675 |  |  |
| 144504 | 144398 |  |  | 2.419 |  |  |  |
| 144795 | 144689 |  | -2.797 |  |  |  |  |
| 146354 | 146248 |  |  | -6.700 |  |  |  |
| 127914 | 127808 |  | -3.202 |  |  |  |  |
| 17367 | 17261 |  |  |  | 5.048 |  |  |
| 25114 | 25008 |  |  | -3.310 |  |  |  |
| 35141 | 35035 |  |  |  | -3.552 |  |  |
| 42183 | 42077 |  |  | 2.523 |  |  |  |
| 53810 | 53704 |  |  | 3.432 |  |  |  |
| 55840 | 55734 |  | -3.475 |  |  |  |  |
| 71822 | 71716 |  |  | 4.343 |  |  |  |
| 75512 | 75406 |  |  | 3.103 |  |  |  |
| 104131 | 104025 | -2.715 |  |  |  |  |  |
| 108412 | 108306 | 3.385 |  |  |  |  |  |
| 124516 | 124410 |  |  |  | -5.272 |  |  |
| 125469 | 125363 |  |  | -2.823 |  |  |  |
| 126639 | 126533 | -2.616 |  |  |  |  |  |
| 130422 | 130316 | 6.346 |  | 6.707 |  |  |  |
| 133177 | 133071 | -3.214 |  |  | 5.892 |  |  |
| 133616 | 133510 |  |  | -2.946 |  |  |  |
| 133688 | 133582 | 3.872 |  | 3.566 |  |  |  |
| 134944 | 134838 |  |  | -2.668 |  |  |  |
| 135627 | 135521 |  |  | 2.860 |  |  |  |
| 137105 | 136999 |  | 3.820 |  | 3.778 |  |  |
| 138275 | 138169 |  |  | -8.119 | -7.358 |  |  |
| 139127 | 139021 |  |  |  | 3.931 |  |  |
| 144440 | 144334 |  |  | 2.481 |  |  |  |

CE = Carbohydrate Esterase; GH = glycoside hydrolase; CBM = Carbohydrate Binding Module; CYP = Cytochrome p450 monooxygenase
